# Prefrontal cortex and putamen grey matter alterations in cannabis and tobacco users

**DOI:** 10.1177/02698811221117523

**Published:** 2022-09-16

**Authors:** Yusuf Daniju, Paul Faulkner, Kaz Brandt, Paul Allen

**Affiliations:** 1 School of Psychology, Whitelands College, University of Roehampton, London, UK; 2 Combined Universities Brain Imaging Centre, Royal Holloway University of London, UK; 3 Psychosis Studies, Institute of Psychiatry, Psychology and Neuroscience, King’s College London, UK

**Keywords:** Cannabis, tobacco, grey matter, prefrontal cortex, putamen

## Abstract

**Background:**

Previous magnetic resonance imaging studies in regular cannabis users report altered grey matter volume (GMV) in brain regions, including the prefrontal cortex (PFC), putamen and hippocampus. However, most studies have tended to recruit recreational users with high levels of cannabis use, and have not controlled for the possible confounding effects of tobacco use. We attempt to address these limitations in the present study.

**Methods:**

We acquired volumetric images in sex, age and IQ-matched groups of (1) regular Cannabis users who also smoke Tobacco cigarettes (‘CT’; *n* = 33), (2) non-cannabis-using Tobacco cigarette smokers (‘T’; *n* = 19) and (3) non-cannabis/tobacco-using Controls (‘C’; *n* = 35). GMV in bilateral PFC, putamen and hippocampal regions was compared across groups. We also examined the associations between GMV differences and levels of cannabis and tobacco use, measures of intellectual function, and of depression, anxiety and stress.

**Results:**

Relative to controls, both CT and T groups showed lower GMV in the left inferior frontal gyrus, and greater GMV in the putamen. In addition, lower GMV in the right frontal pole in the CT group (but not the T group) was associated with lifetime cannabis use, but not with cigarette use.

**Conclusions:**

Regular cannabis users who also smoked tobacco cigarettes showed altered GMV patterns relative to controls. However, a similar pattern of GMV differences was also seen between regular tobacco users that did not use cannabis. Further research is needed to disentangle the effects of cannabis and tobacco use on brain structure.

## Introduction

There are believed to be around 200 million regular cannabis users worldwide (United Nations Office of Drugs and Crime, 2018), and recreational use is likely to increase as cannabis use is decriminalised or made legal in many regions of the world. Thus, investigating the effects of regular cannabis use on the brain is increasingly essential.

Animal studies investigating the main psychoactive substance in cannabis, Δ^9^ tetrahydrocannabinol (THC), demonstrate dose-dependent neurotoxicity in cannabinoid receptor-rich regions of the brain (e.g. Chan et al., 1998; Heath et al., 1980; Lawston et al., 2000). Worryingly, over recent decades, there has been a trend for recreational users to use stronger and more potent strains of cannabis that have increased concentrations of THC that can potentiate the psychoactive effects of cannabis (Bhattacharyya et al., 2010).

Magnetic resonance imaging (MRI) has been used to examine the effects of regular cannabis use on brain structure and volume in humans (e.g. Lorenzetti et al., 2019; see Daniju et al., 2020 for review). Generally, and broadly in line with animal studies, voxel-based morphometry (VBM) studies in human participants have reported lower grey matter volume (GMV) in regular and heavy cannabis users compared to non-cannabis-using control groups, particularly in the prefrontal cortex (PFC) and in the hippocampus (e.g. Ashtari et al., 2011; Battistella et al. 2014; Demirakca et al. 2011; Filbey et al, 2015; Lorenzetti et al, 2015; Yücel et al., 2008), both of which have a high density of endocannabinoid receptors (Downer et al., 2001; Herkenham et al., 1991; Lorenzetti et al., 2015; Matochik et al., 2005; Malone et al., 2010). These cannabis-related GMV decreases may also be cognitively and clinically significant because regular cannabis use has been associated with both cognitive impairments (Crean et al., 2011; Meier et al., 2012) and adverse mental health outcomes (Henquet et al., 2004; Moore et al., 2007), both of which may be linked to neuroanatomical changes as a result of repeated cannabis exposure (Volkow et al., 2016).

Importantly, although the above studies report that cannabis may influence GMV in the PFC and hippocampus, some volumetric studies have failed to find such effects when examining cannabis users and cannabis non-users (e.g. Block et al. 2002; Tzilos et al. 2005). It is possible that the equivocal findings reported by volumetric studies may be due to a range of confounding variables such as other substance usage and the magnitude of lifetime exposure to cannabis (see Bossong et al., 2014).

For example, a potential confounding factor is that the majority of previous volumetric studies did not control for tobacco use across cannabis-using and non-cannabis-using groups (e.g. Cousijn et al., 2012; Yücel et al., 2008). This is problematic as tobacco is often used with cannabis (Banbury et al., 2013; United States Department of Health and Human Services, Substance Abuse and Mental Health Services Administration, 2012), and tobacco use is also associated with relatively lower GMV in PFC regions (Brody et al., 2004; Faulkner et al., 2020; Franklin et al., 2014; Fritz et al., 2014; Gallinat et al., 2006). Currently, only two studies have attempted to determine the contribution of co-occurring cannabis and tobacco use on brain volume (i.e. Filbey et al., 2015; Wetherill et al., 2015). Importantly, Wetherill et al. (2015) report that cannabis, tobacco and cannabis + tobacco users exhibited larger GMV than the non-drug-using controls in the left putamen, indicating that use of both cannabis and tobacco may be associated with changes in GMV in this brain region. Conversely, only cannabis use (but not tobacco use) was associated with larger GMV in the precentral gyrus compared to controls. Filbey et al. (2015) compared GMV within a hippocampal region of interest (ROI), and reported that the use of cannabis, either on its own or in conjunction with tobacco (but not tobacco use without cannabis), was associated with smaller GMV in the hippocampus compared to controls.

Another potentially confounding factor is that some volumetric studies reported GMV differences between control groups and users who smoked cannabis at least five times per week for at least 10 years (Yücel et al., 2008) or users who smoked cannabis on average 28 days per month and had over 62,000 lifetime cannabis smoking episodes (Lorenzetti et al., 2015).

Given the scarcity of volumetric studies in cannabis users that control for tobacco use, we examined GMV in (1) regular cannabis users that also smoke tobacco cigarettes (‘CTs’), (2) non-cannabis-using tobacco cigarette smokers (‘Ts’) and (3) controls who do not use cannabis or tobacco (‘Cs’). Importantly, we sought to recruit recreational cannabis users that report a wide variety of cannabis use to obtain a more representative sample of users than those examined in some previous studies (e.g. Filbey et al., 2015; Wetherill et al., 2015; Yücel et al. 2008).

It was predicted that, relative to controls who do not use cannabis or tobacco (C), regular cannabis users who also smoke tobacco cigarettes (CTs) would show lower GMV in PFC and hippocampal regions, and greater putamen GMV. By recruiting a non-cannabis-using tobacco-smoking group (Ts), we were able to examine whether similar volumetric patterns were observable due to tobacco use only. We also examined whether there was an association between GMV and the levels of lifetime cannabis and tobacco use. Finally, given the link between cannabis use, adverse mental health and intellectual function (Crean et al., 2011; Meier et al., 2012), we explored the relationship between GMV in regions in which there is an effect of group on GMV with a measure of IQ and with levels of depression, anxiety and stress.

## Methods

### Participants

The University of Roehampton Ethics Committee provided ethical approval for the study and all participants gave written informed consent prior to taking part. The cannabis users who also use tobacco (‘CT’) and the tobacco-only users (‘T’) were recruited specifically for this study. Data from the control participants who did not use cannabis or tobacco (C) were collected as part of an ongoing study using identical measures of brain volume (see below), as well as assessing the levels of cannabis and tobacco use. This study also had ethical approval from the Roehampton Ethics Committee. Participants ranged from 18 to 37 years of age (*M* = 22.97 years, standard deviation (SD) = 4.23) and were recruited via online and print advertisements at the University of Roehampton and Royal Holloway University of London.

Participants (subsequently assigned to the CT and T groups) first completed an online survey in Qualtrics (https://www.qualtrics.com) using the Cannabis Experience Questionnaire (Barkus et al., 2006). In all, 129 respondents began the questionnaire, 50 respondents did not complete the questionnaire, and 27 respondents did not meet the inclusion criteria for the study. The remaining 52 respondents were contacted and asked to take part in the study. Exclusion criteria for the study were a self-reported history of neurological or psychiatric disorders (other than Cannabis and Tobacco Use Disorders), current drug dependency or drug use within 6 months of MRI scanning, other than for cannabis or tobacco dependency, and any contraindications for MRI. All participant demographic, cannabis and tobacco use data are shown in Table 1.

**Table 1.** Mean (SD) demographic, cannabis and tobacco use across CT, T and C groups.

	Tobacco users (T) *N* = 19	Cannabis and tobacco users (CT) *N* = 31	Non-smoking Controls (C) *N* = 35	Analysis
Male/female	5/14	18/13	15/20	*X*^2^ = 3.28 *p* = 0.06
Age (years)	22.8 (3.6)	23.3 (3.7)	22.8 (4.9)	*F*(2,82) = 0.227 *p* = 0.75
Mean IQ	105.6 (10.88)	109.2 (11.35)	111.54 (9.69)	*F*(2,82) = 1.78 *p* = 0.18^a^
Daily cigarettes smoked	6.6 (5.3)	4.8 (5.5)	0	*t*(45) *=* 1.58 *p* = 0.12^b^
Years of tobacco use	6.2 (4.2)	5.3 (4.3)	0	*t*(45) = 1.95 *p* = 0.35^b^
Pack years	2.7 (3.65)	2.1 (4.07)	0	*t*(45) *=* 1.02 *p* = 0.31^b^
Total lifetime joints	25.1 (43.0)	3703 (4465)	0	*t*(45) = 12.10 *p* = 0.001
Years of cannabis use	0	8.2 (4.4)	0	n/a

SD: standard deviation; WASI: Weschler Abbreviated Scale of Intelligence; WRAT: Wide Range Achievement Test.

a WASI in CT and T groups, WRAT in C group.

b Test between CT and T groups only.

### Cannabis/tobacco users (CT group)

For the purpose of capturing a broad spectrum of social and recreational cannabis use, cannabis use was defined as ‘the use of at least one cannabis joint per week for at least 6 months prior to MRI scanning’. In all, 33 regular cannabis users were recruited to the study. Of these, 20 were daily users of cannabis, while the remaining 13 were intermittent users who self-reported cannabis use ranging from 2 to 3 joints a week. For the CT group, the mean number of weekly cannabis smoking sessions was 4.5 (SD = 2.2), with a mean number of 1.93 (SD = 1.4) joints per session. The mean duration that participants in the CT group had smoked cannabis was 8.2 years (SD = 4.5, range = 0.07–12.2). Total lifetime joints were calculated as the average number of joints smoked per session (1.93), multiplied by the number of cannabis smoking sessions per week (4.5 × 52 weeks) multiplied by the number of years (8.2) as a cannabis user. As such, the mean total lifetime cannabis joints smoked in the ‘CT’ group were 3703.28 (SD = 4465). Participants in the CT group also smoked tobacco, the mean number of tobacco cigarettes smoked per day by participants in this group was 5.34 (SD = 6.28, range = 1–20). Participants were asked to abstain from cannabis use for 24 h before MRI scanning. One participant in the CT group was removed as they reported that they did not follow this instruction to abstain from cannabis use for 24 h before the MRI scan. Another cannabis user was removed as an outlier as their total lifetime cannabis use was more than 2 SDs above the mean (3703.28), meaning that analyses were performed on data from 31 regular cannabis users who also smoked tobacco.

### Tobacco users (T group)

Initially, 21 non-cannabis-using tobacco cigarette smokers were recruited to the study. Participants in the ‘T’ group smoked at least one tobacco cigarette per week with a mean of 6.6 cigarettes per day (SD = 5.3). ‘Pack years’ (mean = 2.75, SD = 3.65, range = 0.2–144) was calculated as the average number of packs of cigarettes smoked per day multiplied by the number of years of smoking, as in previous research (e.g. Durazzo et al., 2016; Gallinat et al., 2006; Faulkner et al., 2020). Tobacco users had limited lifetime exposure to cannabis, with no self-report history of regular use and no use reported 6 months prior to MRI scanning. The mean number of lifetime cannabis joints for the tobacco-using group = 25.10 (SD = 43.00). Two of the T group were excluded due to a prior history of regular cannabis, who did not meet criteria for the CT group. The final ‘T’ group size was *N* = 19.

### Individuals who do not use cannabis or tobacco (C group)

In all, 35 participants who reported smoking fewer than 10 cigarettes in their lifetime and no cannabis use (recruited for the separate study) were included in this dataset; importantly, they were all matched for age, sex and estimated IQ with the participants in the ‘CT’ and ‘T' groups. Participants for this study were recruited from the participant recruitment systems of both Roehampton University and Royal Holloway University, and by word of mouth. Participants had no prior history of neurological illness or contraindications for MRI scafnning.

### Psychometric measures

#### Depression Anxiety Stress Scale (DASS-21; Lovibond and Lovibond, 1995)

The DASS is a self-report questionnaire consisting of 21 items to measure negative affect (depression, anxiety and stress) with seven items per subscale. The DASS-21 has been shown to have good construct validity (Henry and Crawford, 2005). Participants are asked to score every item on a scale from 0 (did not apply to me at all) to 3 (applied to me very much).

##### IQ measures

For the ‘CT’ and ‘T’ groups, participants undertook the Weschler Abbreviated Scale of Intelligence (WASI: Wechsler, 2011). This provides a measure of performance and verbal IQ from which full-scale IQ is then calculated. The mean full-scale IQ for the ‘CT’ group was (*M* = 109.28, SD = 11.35) and for the ‘T’ group was (*M* = 105.60, SD = 10.88). For the ‘C’ group, WASI data were not available. Instead, an estimated measure of full-scale IQ was obtained using the Wide Range Achievement Test (WRAT: Jastak and Wilkinson, 1984) reading Level 2 (*M* = 111.54, SD = 9.69).

## MRI volumetric scan acquisition

MRI scanning was performed on a 3 Tesla Siemens Magnetom TIM Trio Scanner using a 32-channel head coil at the Combined Universities Imaging Centre (http://www.cubic.rhul.ac.uk/). Structural T1-weighted Magnetization Prepared Rapid Acquisition Gradient Echo images were acquired in all participants with a spatial resolution of 1 mm^3^ in plane resolution of 256*256*176 slices with a scanning time of approximately 5 min. Head movement was reduced by cushioning the participants in the head coil with padding. For data collection of the T1-weighted structural scans, the participants were instructed to lie still inside the scanner with the option to open or close their eyes.

## MRI volumetric data processing

Volumetric data were pre-processed using the Computational Anatomy Toolbox, (CAT12; http://www.neuro.uni-jena.de/cat/) a toolbox in SPM12 (https://www.fil.ion.ucl.ac.uk/spm/software/spm12/). The T1-weighted images were skull stripped and normalised to the standard SPM tissue probability map. For image spatial registration, the data were registered using the Shooting registration method (Ashburner and Friston, 2011). After this, the images were segmented into grey matter, white matter and cerebrospinal fluid. CAT12 provides a quality control report for each of the scans, after manual inspection of the report file, the images were smoothed using an 8 mm Gaussian Kernel to improve signal to noise ratio.

Voxel-based inferential statistics were performed on the smoothed grey matter images using a random effects model in SPM 12. Age, gender and total intracranial volume were included as regressors of no interest to control for the effects of these variables on regional GMV. Statistical thresholds were initially applied at *p* < 0.05 after family wise error (FWE) correction level for multiple comparisons with bilateral hippocampal, PFC (BA 8, 9, 10, 11, 44, 45, 46) and putamen ROIs, specified using WFU Pickatlas Toolbox (https://www.nitrc.org/projects/wfu_pickatlas). After a significant F-test, to test our a priori hypotheses, we then conducted post-hoc paired group comparisons, (i) CTs versus Cs (ii) Ts versus Cs and (iii) CTs versus Ts. A Bonferroni correction was performed to correct for the three separate post-hoc tests, meaning that effects were only considered to be significant if the *p* value of the peak voxel within a cluster was <0.016 FWE.

VBM parameter estimates for significant clusters identified by post-hoc tests (see section ‘Results’) were extracted and analysed using IBM SPSS Statistics, Version 26. Linear regression tests were performed for significant peaks (identified by group tests) to assess whether the number of lifetime joints and the average number of cigarettes smoked per day were significant predictors of grey matter parameter estimates extracted.

We also explored the associations between parameter estimates and IQ as quantified from the WASI, and depression, anxiety and stress as quantified using the DASS. A Bonferroni correction was also applied to correct for the multiple correlation tests performed, meaning that the results were only considered significant if the relevant *p* < 0.01.

## Results

### Participant demographics, IQ, cannabis and tobacco use

Demographic characteristics, IQ and levels of tobacco and cannabis use are shown for all groups in Table 1. Groups did not differ in terms of age or sex. Furthermore, there was no significant difference between groups in terms of IQ scores (CT mean = 109.2, SD = 11.35; T mean = 105.60, SD = 10.88; HC mean = 111.5, SD = 9.7; *F*(2,82) = 1.78, *p* = 0.18). Importantly, the CT and T groups did not differ in terms of the average number of tobacco cigarettes smoked per day (CT mean = 4.84, SD = 5.49; T mean = 6.55, SD = 5.29; *t*(45) = 1.58, *p* = 0.12) nor pack years (CT mean = 2.10, SD = 4.07; T mean = 2.70, SD = 3.65; *t*(45) = 1.02, *p* = 0.31). By design, the CT group had significantly higher levels of cannabis use (total lifetime joints) than both the T and C groups (Table 1).

### Depression, anxiety and stress

Mean DASS depression, anxiety and stress scores are shown in Table 2. One-way analysis of variance (ANOVA) revealed no significant effects of group for DASS total, depression, anxiety or stress scores (*p* > 0.05 for all tests) across three groups.

**Table 2.** Mean (SD) DASS subscales across CT, T and C groups.

DASS subscale	CT group		T group		C group		Analysis
	*M*	SD	*M*	SD	*M*	SD	
Depression	7.61	(5.95)	8.05	(8.84)	6.64	(8.50)	*F*(2, 82) = 0.29 *p* = 0.75
Anxiety	6.10	(5.05)	6.26	(5.30)	6.17	(7.00)	*F*(2, 82) = 0.005 *p* = 0.99
Stress	9.71	(7.24)	9.74	(7.87)	11.17	(9.60)	*F*(2, 84) = 0.306 *p* = 0.74

DASS: Depression Anxiety Stress Scale; SD: standard deviation.

### Regions of interest: grey matter volumes

#### Bilateral hippocampal ROI

A one-way ANOVA omnibus test revealed a non-significant effect of group (no supra-threshold effect at *p* (peak) < 0.05 FWE) in the bilateral hippocampal ROI.

No further tests were performed with hippocampal GMV as all group effects were non-significant in this ROI.

#### Bilateral putamen ROI

**Figure 1.**
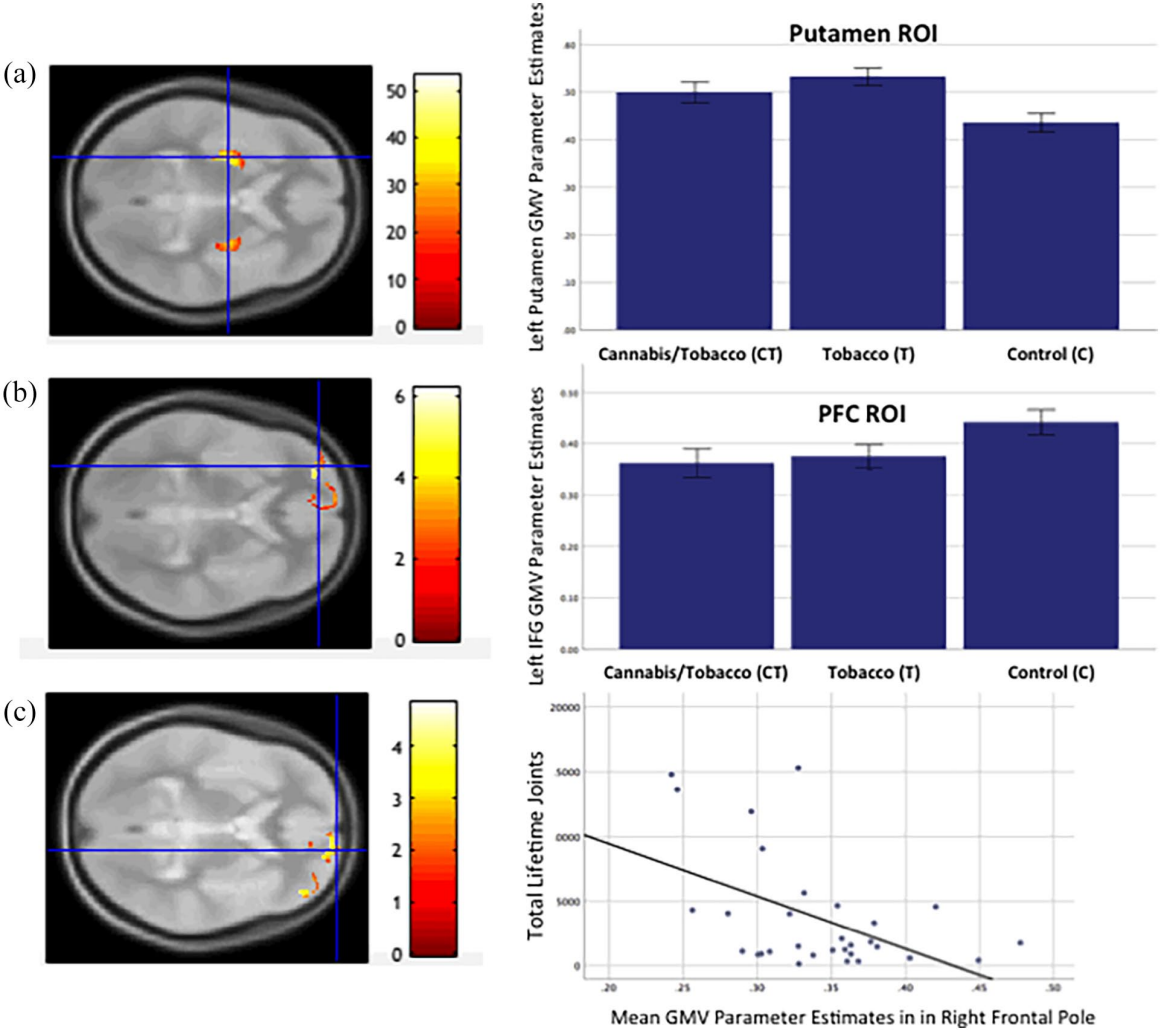
(a) SPM showing Group effect (axial orientation) in bilateral putamen ROI (CT > C) and left putamen (T > C) (*p* = 0.0001 unc. for illustration). Bar chart shows GMV parameter estimates by group in putamen peak. **(**b) SPM showing Group effect (axial orientation) in bilateral PFC ROI (CT and T > C) in left inferior frontal (CT > C) (*p* = 0.001 unc. for illustration). Bar chart shows GMV parameter estimates by group in left inferior frontal gyrus. (c) SPM showing Group effect (axial orientation) in bilateral PFC ROI (CT > C) and right frontal pole (CT > C) (*p* = 0.001 unc. for illustration). Scatterplot showing right frontal pole parameter estimates against total lifetime joints in the CT group. GMV: grey matter volume; PFC: prefrontal cortex; ROI: region of interest.

A one-way ANOVA omnibus test revealed a significant effect of group in the bilateral putamen ROI (*x* = −28, *y* = −9, *z* = 8, *Z* = 7.8, *k* = 927, *p* FWE (peak) < 0.01, and *x* = 30, *y* = −6, *z* = 3, *Z* = 6.54, *k* = 444, *p* (peak) < 0.01; see Figure 1(a)). To test our a priori hypotheses, we conducted the following post-hoc-tests:

CT versus C: Relative to the non-drug-using controls, the cannabis users who also smoked tobacco cigarettes showed greater GMV in left (*x* = −28, *y* = −9, *z* = 8, *Z* = 9.93, *k* = 1639, *p* FWE (peak) = 0.001) and right putamen (*x* = 30, *y* = −8, *z* = 4, *Z* = 6.5, *k* = 1069, *p* FWE (peak) = 0.001). There were no regions within the bilateral putamen ROI where the cannabis users exhibited lower GMV relative to the controls (no suprathreshold effects).

T versus C: Relative to the non-drug-using controls, the tobacco-only users showed greater GMV in the left putamen (*x* = −28, *y* = −8, *z* = −6, *Z* = 5.69, *k* = 433, *p* FWE (peak) = 0.015) but not the right putamen. There were no regions within the putamen ROI where the tobacco-only users showed lower GMV relative to the controls (no suprathreshold effects).

CT versus T: Comparing cannabis users who also smoked tobacco with the tobacco-only users revealed no significant differences in GMV in the putamen ROI (no suprathreshold effects).

#### Prefrontal cortex ROI

A one-way ANOVA revealed a significant effect of group in the left inferior frontal gyrus (*x* = −30, *y* = 51, *z* = 0, *Z* = 5.77, *k* = 59, *p* FWE (peak) < 0.01; see Figure 1(b)). To test our a priori hypotheses, we conducted the following post-hoc tests:

CT versus C: Relative to the non-drug-using controls, the cannabis users who also used tobacco showed lower GMV in the left inferior frontal gyrus (*x* = −30, *y* = 51, *z* = 0, *Z* = 5.54, *k* = 77, *p* FWE (peak) = 0.001), and in the right frontal pole (*x* = 15, *y* = 63, *z* = −3, *Z* = 4.90, *k* = 13, *p* FWE (peak) = 0.001) (see Figure 1(c)). There were no PFC regions where the cannabis users showed greater GMV relative to the control group (no suprathreshold effects).

T versus C: Relative to the C group, the T group exhibited reduced GMV in the left inferior frontal gyrus (*x* = −30, *y* = 51, *z* = −2, *Z* = 5.21, *k* = 11, *p* FWE (peak) = 0.002) (Figure 1(c)). There were no PFC regions where the tobacco-only group showed greater GMV relative to the control group (no suprathreshold effects).

### Associations between right frontal pole GMV, cannabis and tobacco use

As lower right inferior frontal pole GMV was observed in the CT group only, regression analysis was performed within the CT group to establish if there was a dose relationship between cannabis and or tobacco use and volumetric change in this region. There was a significant negative correlation between GMV in the peak voxel within the right frontal pole identified by the post-hoc test CTs < Cs and total lifetime joints (*r* = −0.49, *p* = 0.005) (Figure 1(c)). Linear regression shows that total lifetime joints, β = −0.43, *t*(25) = −2.48, *p* = 0.02, was a significant predictor of grey matter parameter estimates in this region, but that the average number of tobacco cigarettes smoked per day was not (β = −0.24, *t*(25) = −1.43 *p* = 0.16).

### Associations between GMV, DASS and IQ scores

DASS and IQ correlations across PFC and putamen ROI for CT and T groups are shown in Table 3. No tests were performed with hippocampal GMV as all group effects were non-significant in this ROI. There was a significant positive correlation between GMV in the right frontal pole cluster observed in Figure 1(c) and IQ scores in the CT group (*r* = 0.401, *p* = 0.02); however, this association was not statistically significant after Bonferroni correction for multiple tests (*p* > 0.01). All other tests were non-significant.

**Table 3.** Bivariate correlations (two-tailed) between IQ, DASS subscales and GMV parameter estimates from significant group effect peaks in CT and T groups.

	Right frontal pole	Left inferior frontal gyrus	Left putamen	Right putamen
CT group				
IQ	*r* = 0.40, *p* = 0.02	*r* = 0.20, *p* = 0.27	*r* = 0.09, *p* = 0.67	*r* = –0.31, *p* = 0.08
DASS-D	*r* = 0.19, *p* = 0.29	*r* = –0.21, *p* = 0.27	*r* = –0.33, *p* = 0.10	*r* = –0.17, *p* = 0.37
DASS-A	*r* = –0.14, *p* = 0.43	*r* = –0.17, *p* = 0.36	*r* = –0.29, *p* = 0.21	*r* = 0.18, *p* = 0.33
DASS-S	*r* = –0.18, *p* = 0.32	*r* = –0.16, *p* = 0.20	*r* = 10, *p* = 0.76	*r* = –0.05, *p* = 0.75
T group				
IQ	**_**	*r* = –0.26, *p* = 0.28	*r* = –0.15, *p* = 0.25	**_**
DASS-D	**_**	*r* = –0.10, *p* = 0.67	*r* = 0.22, *p* = 0.23	**_**
DASS-A	**_**	*r* = 0.32, *p* = 0.21	*r* = 0.18, *p* = 0.30	**_**
DASS-S	**_**	*r* = 0.32, *p* = 0.18	*r* = 0.20, *p* = 0.26	**_**

DASS: Depression Anxiety Stress Scale; GMV: grey matter volume.

Note, as there were no group effects in the right frontal pole or right putamen for T versus C groups, we did not conduct correlational analysis in the T group.

## Discussion

The current study investigated whether there were volumetric differences within the bilateral hippocampus, PFC and putamen between regular cannabis users who also smoke tobacco cigarettes and both (a) tobacco cigarette-smokers who do not use cannabis and (b) controls who do not use either cannabis or tobacco. Our analyses revealed that, relative to the control group, participants who used both cannabis and tobacco cigarettes exhibited significantly lower GMV in the left inferior frontal gyrus and the right frontal pole. Furthermore, relative to the control group, users of both cannabis and tobacco showed greater GMV in the bilateral putamen. Interestingly, participants who used only tobacco also showed a similar pattern of lower GMV in the left inferior frontal gyrus and greater GMV in the left putamen compared to the controls. However, this tobacco-only group did not show significantly lower GMV in the right frontal pole or greater GMV in the right putamen relative to controls. Contrary to our prediction, GMV in the bilateral hippocampal ROI did not differ between the control, CT and T groups.

That cannabis and tobacco user group exhibited lower GMV within the PFC relative to controls and is generally consistent with findings from previous studies that have examined GMV in cannabis users (e.g. Battistella et al., 2014; Lorenzetti et al., 2015). However, a similar pattern of reduced GMV within the left inferior frontal gyrus was also seen in T group compared to the controls. While previous volumetric studies have also reported that tobacco use is associated with lower than normal GMV within PFC regions (e.g. Faulkner et al., 2020; Franklin et al., 2014; Fritz et al., 2014), this is the first study to report similarly low PFC volume in both cannabis users who also use tobacco, and non-cannabis-using tobacco smokers. This finding raises the possibility that the low GMV within the PFC is not entirely attributable to the neurotoxic effects of cannabis, but may be partly due to regular tobacco use. However, we also observed an area of reduced GMV in the right frontal pole in the CT group that was not seen in T group, and, lower GMV in this right frontal pole region was associated with total lifetime joints used but not the mean number of cigarettes smoked per day. This suggests that while tobacco use may be associated with low GMV in some PFC regions, regular cannabis and tobacco use may be associated with a more widespread pattern of low PFC GMV. As such, future studies may wish to test this hypothesis.

Relative to the control group, cannabis users who also smoked cigarettes exhibited greater GMV in the bilateral putamen, which is consistent with previous findings by Gilman et al (2014). However, greater putamen GMV was also observed in the tobacco-only group, albeit in the left rather than the bilateral putamen. This finding is broadly consistent with previous reports from a number of earlier studies in tobacco users (e.g. Yu et al., 2011) and by Wetherill et al. (2015) who reported greater putamen GMV in cannabis users that also used tobacco, relative to non-drug-using controls. Interestingly, increased putamen volume has been reported in compulsive groups (Radua and Matiax-Cols, 2009) and increased putamen GMV observed in the present study may be associated with the long-term, compulsive use of cannabis and tobacco. Furthermore, it has been suggested that greater putamen GMV may be present before the onset of substance use and could influence the development of substance use disorders (Ersche et al., 2012). While this broadly fits with the striatum’s role in craving and drug seeking behaviour (Wong et al., 2006), it is important to note that classically, only the ventral region of the neostriatum (nucleus accumbens) seems to be involved in craving, drug seeking behaviour (Dias et al., 2021; Crespo et al., 2022), where no GMV changes were seen in the present study.

Contrary to our hypothesis, no differences in hippocampal GMV were observed between cannabis and tobacco users and non-drug-using controls, despite this being a region that is rich in endocannabinoid receptors and a number of previous studies reporting lower GMV in cannabis users compared to non-users (e.g. Ashtari et al 2011; Battistella et al. 2014; Demirakca et al. 2011; Lorenzetti et al, 2015; Yücel et al., 2008). This may be because the cannabis and tobacco-using group in the current study were relatively young and had lower level of lifetime cannabis use relative to the cohorts recruited in some of those previous studies (i.e. Matochik et al., 2005; Yücel et al., 2008). Thus, even though an association between regular cannabis use and lower GMV within the medial temporal lobes has been reported, it is possible that changes in volume of the hippocampus and medial temporal lobes are associated with heavier and/or longer term cannabis use. Future studies, although logistically difficult to conduct, may wish to determine the longitudinal effects of such cannabis use on hippocampal GMV.

Our results are largely consistent with previous research suggesting that long-term cannabis and tobacco use alters brain volume in regions rich in cannabinoid 1 receptors (Svizenska et al., 2008) and nicotinic acetylcholine receptors (Picard et al., 2013), respectively. However, there are few studies investigating the behavioural, physiological and/or neurotoxic effects of cannabis and tobacco co-use/administration. In terms of sensory and cognitive processing in humans, simultaneous use of cannabis and tobacco may enhance these functions by increasing frontal mismatch negativity (de la Salle et al., 2019). Animal studies have shown that combined inhalation of nicotine and THC resulted in physiological and behavioural effects independent of single drug administration, and such effects were either additive or opposed (Javadi-Paydar et al., 2019). However, to our knowledge, there are no studies investigating the neurotoxic effects of cannabis/THC and tobacco/nicotine co-use/administration on GMV in humans or animals. Interestingly, there is emerging evidence that additional molecular targets for cannabinoids exist other than cannabinoid receptors, and that these targets may represent important novel sites to alter neuronal excitability or physiological effects of cannabis and tobacco co-use (Oz et al., 2014). Future work is therefore needed to understand potential, additive, synergistic or opposing effects of cannabis and tobacco co-use.

### Limitations

There are several limitations to the current study. First, we were unable to recruit a cannabis-only-using group (i.e. cannabis smokers that do not use tobacco). This means a ‘clean’ disassociation between the effects of cannabis and tobacco use on brain volume was not completely possible. A further limitation was the self-report procedure used to determine current/recent cannabis use. Cannabis use in the CT group was not confirmed using urine screening. However, urine screening can typically only detect cannabis that was consumed in the previous 7–10 days and would therefore not confirm regular use over a longer duration. Hair analysis techniques that allow more detailed assessment of substance use over time were not available to the research team. Furthermore, quantifying cannabis and tobacco use via measures of ‘lifetime joints smoked’ and ‘pack years’ (for tobacco), although used widely used in previous studies, does not take into account other forms of cannabis and tobacco/nicotine consumption, such as edible products and electronic vaping. Future studies should aim to quantify cannabis and tobacco use across a broader spectrum of consumables.

Another limitation with the current study is that the tobacco use-only group (i.e. ‘T’ group) (*n* = 19) was smaller than the group containing users of both cannabis and tobacco (*n* = 31) and the control group (*n* = 35). Our tobacco-only group also used limited amounts of cannabis in the past, although this was an extremely low level relative to the CT group (i.e. total lifetime joints = 25.1 vs. 3703 in our CT group). Furthermore, no participants included in the T group analysis reported using cannabis in the 6 months prior to MRI scanning. However, future work should endeavour to have bigger and equal-sized groups to improve the power, and to also include a group that uses cannabis but not tobacco. Finally, WASI data were not available for participants in the C group. Instead an estimate of full-scale IQ was used, that is, the WRAT (Jastak and Wilkinson, 1984). However, the WRAT provides an IQ estimate that is reported to be highly correlated with full-scale IQ measured by the Wechsler Adult Intelligence Scale (Jastak and Wilkinson, 1984), and in the current study we felt it was important to establish whether groups were matched for intellectual function. Furthermore, for correlation and regression analyses within CT and T groups, exploring associations between IQ, GMV and substance use variables, only WASI data were used.

In summary, the results from this study indicate that, in a young adult population of regular cannabis and tobacco users who exhibit a range of recreational cannabis use patterns, cannabis and tobacco use is associated with lower than normal GMV within the PFC and putamen. This is in line with a previous study that reports volumetric changes in young people with limited exposure to cannabis (Orr et al., 2019). However, similar volumetric alterations were also observed in non-cannabis-using tobacco smokers, and further work is therefore needed to better understand the differential effects of regular cannabis and tobacco use on brain volume.
